# UBAC2 promotes bladder cancer proliferation through BCRC-3/miRNA-182-5p/p27 axis

**DOI:** 10.1038/s41419-020-02935-7

**Published:** 2020-09-10

**Authors:** Chaohui Gu, Keyuan Zhao, Naichun Zhou, Feng Liu, Fei Xie, Shunli Yu, Yongjie Feng, Long Chen, Jinjian Yang, Fengyan Tian, Guosong Jiang

**Affiliations:** 1 Departments of Urology, Henan Institute of Urology and Zhengzhou Key Laboratory for Molecular Biology of Urological Tumor Research, The First Affiliated Hospital of Zhengzhou University, Zhengzhou, Henan 450052 China; 2 Department of Urology, Union Hospital, Tongji Medical College, Huazhong University of Science and Technology, Wuhan, 430022 China; 3 Department of Urology, The Affiliated Hospital of Qingdao University, Qingdao, 266013 China; 4 Departments of Pediatrics, The First Affiliated Hospital of Zhengzhou University, Zhengzhou, Henan 450052 China

**Keywords:** Oncogenes, Oncogenes, Oncogenes, Bladder cancer, Bladder cancer

## Abstract

Emerging evidences have demonstrated that ubiquitin-associated domain-containing protein 2 (UBAC2) is closely related to the occurrence and development of malignant tumors. However, the functions and underlying molecular mechanisms of UBAC2 in bladder cancer (BC) development have not been defined. In this study, we found that both UBAC2 mRNA and protein levels were upregulated in BC tissues and cell lines, and knockdown of UBAC2 inhibited BC cells proliferation both in vitro and in vivo. Meanwhile, Kaplan–Meier survival plots of 406 BC cases from TCGA database showed that higher expression of UBAC2 in BC patients was associated with lower survival rate. Mechanistic studies revealed that knockdown of UBAC2 increased the expression of p27 by posttranscriptional regulation. Our previous study indicated that circular RNA BCRC-3 (BCRC-3) promoted the expression of p27 through interacting with miR-182-5p, and reversed miR-182-5p-induced inhibition of p27 3′UTR activity. In the present study, we found that UBAC2 could bind to BCRC-3, and subsequently affected the interaction of BCRC-3 with miR-182-5p to inhibit the expression of p27. Furthermore, knockdown of BCRC-3 partly reversed the upregulation of p27 expression induced by knockdown of UBAC2. Our findings highlight a novel mechanism of UBAC2 in regulating p27 through affecting the function of BCRC-3, and provide a research basis for the diagnostic and therapeutic application of BC.

## Introduction

Bladder cancer (BC) is a common cancer in the world, with a high incidence and mortality rate^1^. In the United States, there were 80,470 (61,700 men; 18,770 women) new cases of BC and 17,670 deaths (12,870 men; 4800 women) by BC in 2019^2^. The diagnosis and treatment technologies, including surgery, chemotherapy, and immunotherapy have been improved continuously^3,4^. However, the overall survival rate is still very low owing to various clinical characteristics and treatment responses caused by the complex pathogenesis of BC^5^. Therefore, further exploration of the regulatory mechanism involved in BC progression is necessary for the progress of diagnostic markers and novel effective therapies for BC patients.

The ubiquitin-associated domain-containing protein 2(UBAC2) gene is located on chromosome 13q32.3 and encodes UBAC2 protein that is a highly conserved protein in different species^6,7^. It was reported that the polymorphism of UBAC2 gene was related to Behcet’s disease and UBAC2 gene was the risk allele of Behcet’s disease^8–11^. The upregulation of UBAC2 expression can promote the progress of Behcet’s disease^11^. Recently, a growing body of evidences demonstrated that UBAC2 was also closely related to the occurrence and development of malignant tumors, such as skin cancer and BC^12,13^. Moreover, it was reported that the expression level of UBAC2 was diverse in different types and pathological grades of BC by using microarray^13^. However, the specific role, regulatory mechanisms and the upstream regulator/downstream effectors of UBAC2 in human BC are still unclear.

Circular RNAs (circRNAs), a new member of noncoding RNAs (ncRNAs), have been discovered about 40 years by electron microscopy^14,15^. Previously, circRNAs were thought as by-products of splicing errors^16^. However, the development of the high-throughout sequencing and bioinformatic analysis have identified and proved thousands of circRNAs in diverse species^17–19^. Moreover, a lot of studies have demonstrated that circRNAs play crucial roles in many diseases such as neurological disorders, atherosclerotic vascular disease, carcinomas, and so on^20–22^. Recently, increasing numbers of studies indicated that circRNAs could affect the functions of proteins via direct interactions with them^23^. For instance, circRNAs could facilitate their nuclear or cytoplasmic localizations^24,25^, regulate their functions or stability^26,27^, promote or inhibit the interactions between them^21,28^, or influence the interactions between them and DNA by binding directly with proteins^29,30^. The biological roles of RNA–protein interactions are pervasive through direct or indirect interactions^31^. However, little is known about the effect of proteins on the function of circRNAs.

In this research, we explored the expression level and potential role of UBAC2 in BC and found that UBAC2 was overexpressed and knockdown of UBAC2 inhibited BC cell lines proliferation by promoting the expression of p27 in vitro and in vivo. In our recent study, we have demonstrated that BC related circRNA-3 (BCRC-3) can promote the expression of p27 through acting as “miRNA sponge” for miR-182-5p in BC cells^32^. Our further analyses indicated that UBAC2 could increase the expression of p27 through affecting the interaction of BCRC-3 with miR-182-5p in BC cells and then affected the progression of BC. Collectively, we found a novel mechanism of UBAC2 affecting the function of BCRC-3, and UBAC2 may serve as a novel promising target for diagnosis and therapy of BC.

## Materials and methods

### Patient tissue specimens and cell lines

All 48 specimens of BC tissues and their adjacent normal tissues were obtained from patients undergoing radical cystectomy at the Department of Urology of Union Hospital affiliated of Tongji Medical College. Human specimen collection was approved by the Institutional Review Board of Tongji Medical College of Huazhong University of Science and Technology. All tissues were confirmed and classified by more than one experienced pathologist according to the 2004 World Health Organization Consensus Classification and Staging System for bladder neoplasms. Clinicopathological features of patients are listed in Table S1. The human BC cell lines (RT4, EJ, UMUC3, and T24) and human normal urothelial cell line (SV-HUC-1) were obtained from American Type Culture Collection (ATCC, Manassas, VA, USA). The human BC cell line T24T was provided by Dr. Dan Theodorescu (Departments of Urology, University of Virginia, Charlottesville, VA). All cell lines were cultured at 37 °C and 5% CO_2_ with RPMI-1640 medium (Gibco, Grand Island, NY, USA) plus 10% fetal bovine serum (Gibco, Australia origin) and 1% penicillin–streptomycin (Gibco).

### RNA extraction and PCR assays

Total RNA of tissue samples and cell lines were extracted by TRIzol reagent (Invitrogen, Carlsbad, CA, USA) following the manufacturer’s instructions. Complementary DNA synthesized using the HiScript III RT SuperMix for quantitative polymerase chain reaction (qPCR) (Vazyme Biotech, Nanjing, China) with random or oligo(dT) primer. Then, the quantitative real-time PCR (qRT-PCR) was performed using SYBR Green Master Mix (Vazyme Biotech). β-actin or U6 was used as internal control when calculation using the ΔΔCt method. All data were analyzed using the StepOnePlus Real-Time PCR System (Applied Biosystems, Carlsbad, CA, USA). All primers were obtained from RiboBio (Guangzhou, China). The primers are listed in Table S3.

### Western blotting analysis

Total protein was extracted from RIPA lysis buffer (Invitrogen) and the concentration was determined using BCA Protein Assay Kit (Beyotime, Shanghai, China). The protein samples were separated by 10% sodium dodecyl sulfate polyacrylamide gel electrophoresis (SDS-PAGE) gels and then transferred onto polyvinylidene difluoride membranes. After blocked for 1 h at room temperature, membranes were probed with primary antibodies overnight at 4 °C. Then, membranes were incubated for 1 h in the specific horseradish peroxidase (HRP)-conjugated secondary antibodies at room temperature. All images were developed by ECL kit (Servicebio, Wuhan, China) and obtained by using BioSpectrum600 Imaging System (UVP, CA, USA). Antibodies against UBAC2 (Cat No 25122-1-AP) were purchased from Proteintech Group (Wuhan, China). Antibodies against CDK2 (Cat. No. 10122-1-AP), CDK4 (Cat. No: 11026-1-AP), CDK6 (Cat. No:14052-1-AP), cyclin D1 (Cat. No. 60186-1-Ig), Cyclin E (Cat. No. 11554-1-AP), p21 (Cat. No. 10355-1-AP), p27(Cat. No. 25614-1-AP), β-actin (Cat. No. 60008-1-Ig), HRP-conjugated secondary goat anti-mouse (Cat. No. SA00001-1) and goat anti-rabbit (Cat. No. SA00001-2) were purchased from Proteintech Group (Chicago, USA).

### Plasmids construction and stable transfection

The shRNAs targeting UBAC2 (shUBAC2) and BCRC-3 (shBCRC-3) were synthesized by Genechem (Shanghai, China), and were cloned into GV298 vector (Table S4). ShRNAs targeting p27 were obtained from our previous study^32^. The plasmids were transfected into cells by using Lipofectamine 2000 (Life Technologies) according to the manufacturer’s instructions. The transfected cells were selected with puromycin (Invitrogen) for 3–4 weeks. The sequence of shRNAs targeting UBAC2 and BCRC-3 are shown in Table S3.

### Immunohistochemistry analysis

Immunostaining was carried out on BC tissue sections from patients and tumor tissues from xenografts in nude mice, with specific antibodies for UBAC2, p27, and Ki-67 (Cat. No. 27309-1-AP), respectively (Proteintech). An Olympus FSX100 microscope (Olympus, Japan) was used to capture images. The IHC stained sections were evaluated at 400-fold magnification, and 4–6 representative staining fields of each section were analyzed. The protein expression levels were analyzed by calculating the integrated optical density per stained area (IOD/area) using Image-Pro Plus version 6.0 (Media Cybernetics, MD, USA) as described^33^.

### Immunofluorescence (IF) analysis

Immunofluorescence (IF) analysis was carried out as described^34^. Antibody against UBAC2 was purchased from Proteintech Group (Wuhan, China). The FITC-conjugated Goat Anti-Mouse IgG (Cat. No. GB22301) and DAPI obtained from Servicebio (Wuhan, China). All images were obtained using Nikon A1Si Laser Scanning Confocal Microscope (Nikon Instruments Inc., Japan).

### Flow cytometry assay for the cell cycle

EJ and UMUC3 cells stably transfected with plasmids were harvested to analyze cell cycle by flow cytometry (Becton Dickinson, NJ, USA) after stained with propidium iodide buffer (BD Pharmingen). The results were analyzed with ModFit LT software.

### Cell Counting Kit-8 (CCK-8) assay

Cell viability was detected by using Cell Counting Kit-8 assay. Cells stably transfected with plasmids were cultured in 96-well plates until cell confluence reached 60%. Then, a volume of 10 μl of CCK-8 solution(Servicebio) was added to each well at 0, 24, 48, and 72 h. After incubation for 2 h, the absorbance at 450 nm was measured using spectrometer (Thermo Fisher Scientific, MA, USA).

### Colony formation assay

For the colony formation assay, EJ and UMUC3 cells stably transfected with plasmids were seeded in 6-wells plates at density of 600 cells per well and cultured for 2 weeks. The cell colonies were fixed with 4% polyformaldehyde for 30 min and stained with 0.1% crystal violet (Servicebio) for another 20 min at room temperature. Cell colonies with more than 50 cells were counted.

### Tumor xenografts

All animal experiments were approved by the Animal Care Committee of Tongji Medical College (approval no. 20192289). Four-week-old male nude mice from the Institute of Zoology (Beijing, China) were selected for tumor xenografts and randomly divided into two groups (*n* = 5 per group). EJ cells stably transfected with shUBAC2 plasmids or control plasmids were subcutaneously injected into the right flanks of the nude mice (2 × 10^6^, 200 µL), respectively. The volumes of tumors were measured at 1–4 weeks after injection and measured as the length × width^2^ × 0.5.

### RNA immunoprecipitation (RIP) assay

RNA immunoprecipitation (RIP) assay was performed as described^35^. In brief, about 1.5 × 10^7^ cells were harvested and lysed to extract total protein. Total protein was immunoprecipitated with antibody against UBAC2 purchased form Proteintech Group (Cat. No. 25122-1-AP) or IgG antibody as control, and Protein A/G magnetic beads (Life Technologies). Finally, the RNA complexes combining on the protein were purified with RNeasy Mini Kit (QIAGEN, China) for RT-PCR.

### Pull-down assay with biotinylated BCRC-3 probe

Biotinylated-BCRC-3 probe was synthesized by RiboBio. The sequence of the BCRC-3 probe was just complemented to the back-spliced junction of circular BCRC-3 and listed in Table S3. Pull-down assay was performed as our previous study^36^. The RNeasy Mini Kit (QIAGEN, China) was used to extract the bound RNAs for further research.

### Luciferase reporter assay

The p27 3′UTR or promoter reporters were transiently transfected into BC cells together with Renilla control plasmid, concomitantly either with shUBAC2, or shNC. After 24 h, the luciferase activity was determined following dual luciferase reporter assay detection kit (Promega, WI, USA).

### RNA fluorescence in situ hybridization

Cy3-labeled BCRC-3 probe was bought from RiboBio. The assay was performed using fluorescence in situ hybridization kit (RiboBio, Guangzhou, China) according to the manufacturer’s instructions. The signal of the probes was detected by Nikon A1Si Laser Scanning Confocal Microscope (Nikon Instruments Inc., Japan).

### Statistical analysis

All data were analyzed using GraphPad Prism 7.0 (La Jolla, USA). The group difference was evaluated by student’s *t* test or chi-square, presented as means ± standard error of the mean (SEM). Kaplan–Meier survival curve and log-rank test were employed to depict the OS distributions of BC patients with different expression levels of UBAC2. *P* < 0.05 was considered statistically significant.

## Results

### UBAC2 is overexpressed in BC tissues and cell lines

**Fig. 1:**
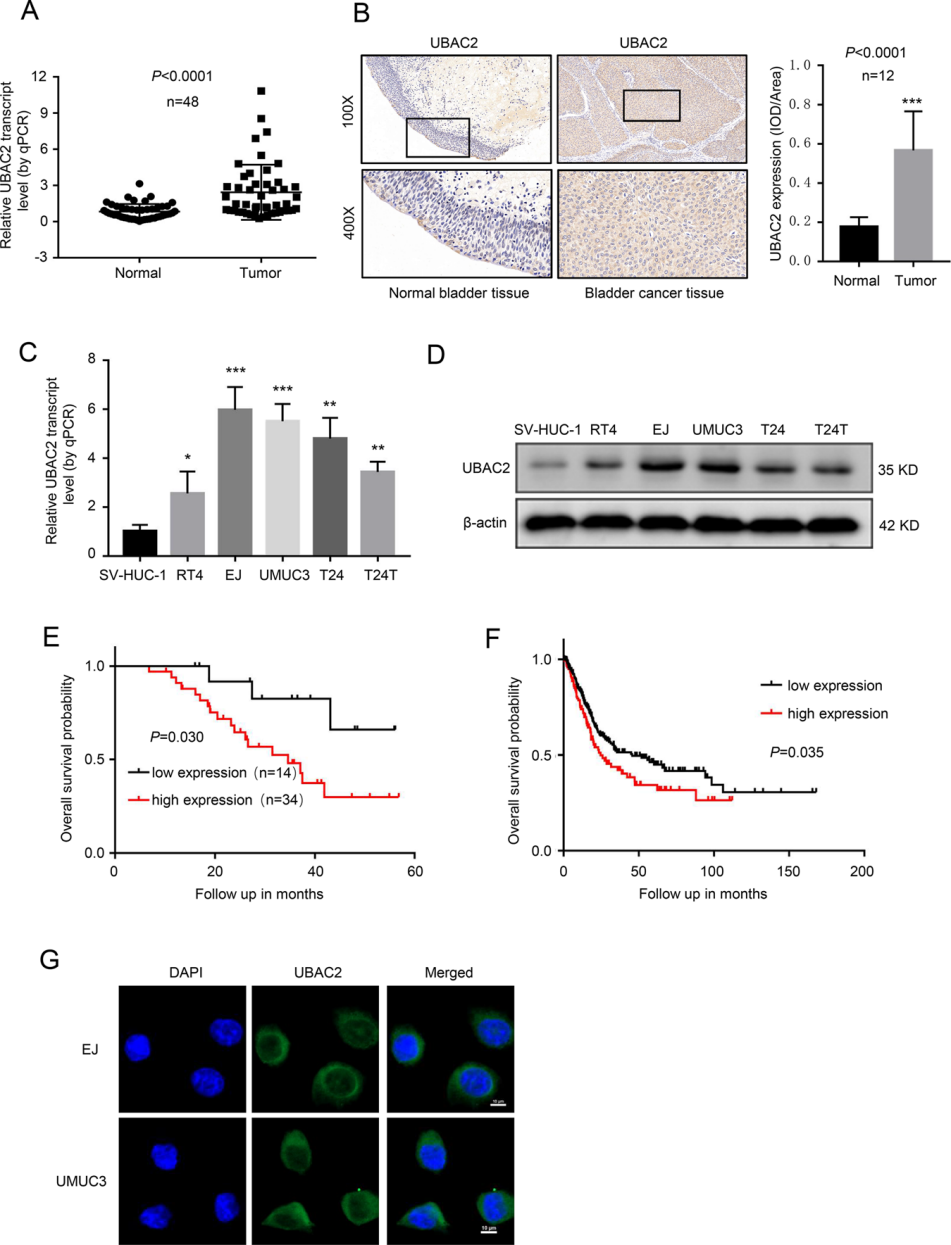
UBAC2 is overexpressed in BC tissues and cell lines. **a** The results from qRT-PCR analysis showed that the mRNA expression levels of UBAC2 were significantly upregulated in BC tissues compared with their adjacent normal tissues. β-actin was used as loading control. **b** The protein expression levels of UBAC2 in 12 samples were determined by immunohistochemistry (IHC) staining and analyzed by calculating the integrated optical density per stained area (IOD/area). The tumor sample shown here is stage T3 with high grade. **c**, **d** The mRNA (**c**) and protein (**d**) levels of UBAC2 in SV-HUC-1, RT4, EJ, UMUC3, T24, and T24T cells were validated by qRT-PCR and western blotting. β-actin was used as loading control. **e** Kaplan–Meier survival plots of 48 bladder cancer cases from our hospital show the survival probability of patients with high or low expression of UBAC2 (*p* = 0.030, log-rank test). **f** Kaplan–Meier survival plots of 406 bladder cancer cases from TCGA database show the survival probability of patients with high or low expression of UBAC2 (*n* = 406, *p* = 0.035, log-rank test). **g** Immunofluorescence (IF) showed that UBAC2 protein stained green was localized in cytoplasm of BC cells. Nuclei were stained blue with DAPI (scale bar, 10 μm). Data are presented as mean ± SEM from three independent replicates. *P < 0.05; **P < 0.01; ***P < 0.001 (Student’s *t* test).

In order to evaluate the expression level of UBAC2 in BC, we analyzed 48 pairs of urothelial carcinoma of bladder tissues and surrounding normal bladder tissues by qRT-PCR assay. As shown in Fig. 1a, the mRNA level of UBAC2 was significantly upregulated in BC tissues. Moreover, immunohistochemistry analysis showed that the expression level of UBAC2 protein was remarkably higher in BC tissues than that in adjacent normal tissues (Fig. 1b). Nevertheless, the expression of UBAC2 had no relationship with BC grade, pathological stage and lymph node metastasis (Tables S1 and 2). Besides, higher expression levels of both UBAC2 mRNA and protein were also detected in RT4, EJ, UMUC3, T24, and T24T BC cell lines as compared with human immortalized uroepithelium cells SV-HUC-1 (Fig. 1c, d). Kaplan–Meier survival plots of 48 BC cases from our hospital (Fig. 1e) and 406 BC cases from TCGA database suggested that patients with higher UBAC2 expression had worse survival probability (Fig. 1f). The images from IF analysis showed that UBAC2 was mainly localized in the cytoplasm of BC cells (Fig. 1g). These results indicated that UBAC2 might promote BC progression.

### Knockdown of UBAC2 represses the proliferation of BC cells in vitro and in vivo

**Fig. 2:**
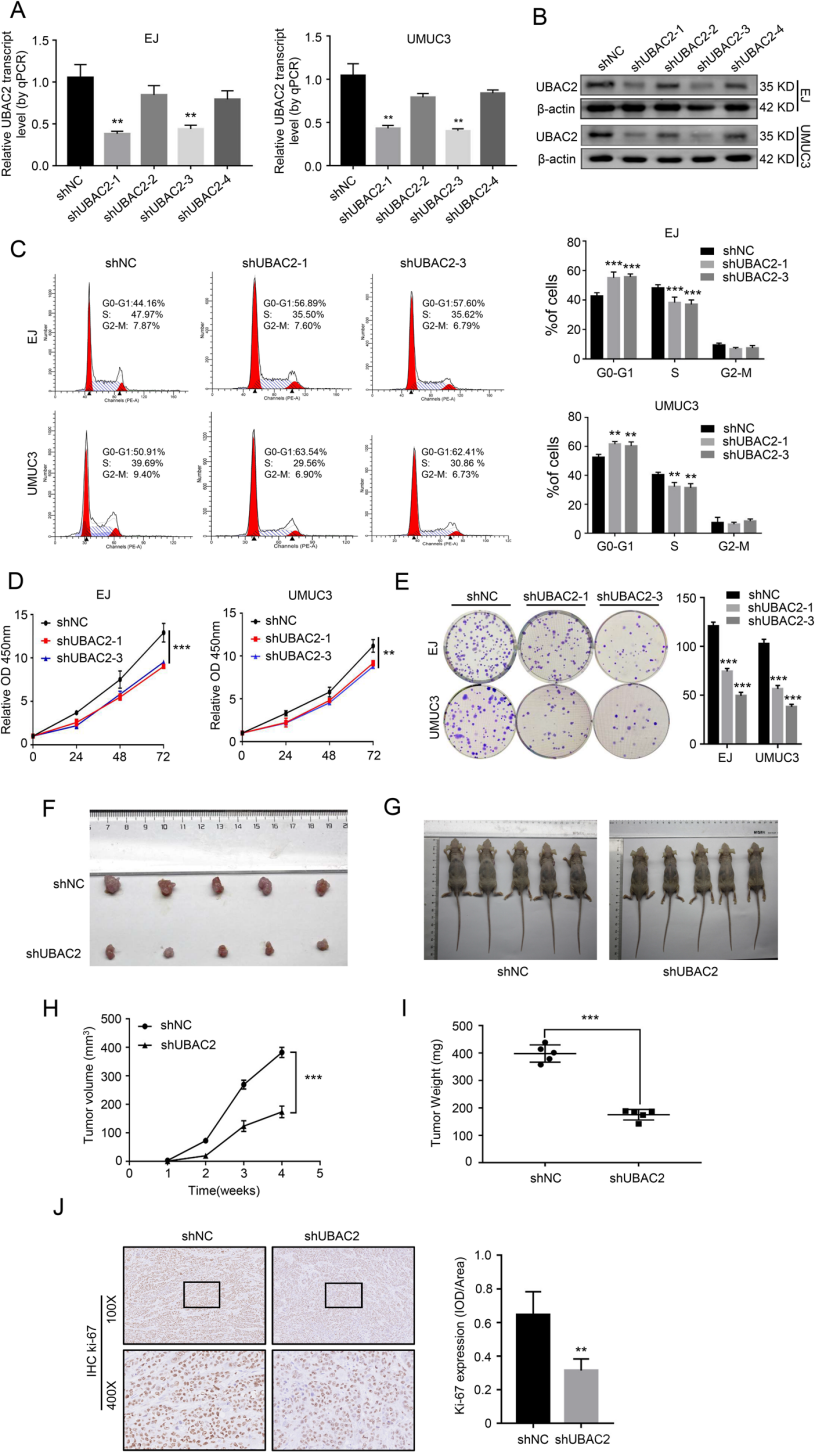
Knockdown of UBAC2 represses the proliferation of BC cells in vitro and in vivo. **a**, **b** The efficiency of UBAC2 knockdown in EJ and UMUC3 cells was detected by qRT-PCR (**a**) and western blotting assay (**b**). β-actin was used as loading control. **c** Flow cytometry indicated that knockdown of UBAC2 resulted in cell cycle arrest at G0/G1 phase in both EJ and UMUC3 cells compared with negative control. **d** Cell viability was evaluated by Cell Counting Kit-8 assay. **e** Plate colony formation assay showed that the colony formation activity was inhibited after shUBAC2 transfection. Colonies with more than 50 cells were counted. **f** Tumors collected from mice were exhibited after 1 month of hypodermic injection. **g** EJ cells stably transfected with shUBAC2 plasmids or control plasmids were subcutaneously injected into the right flanks of the nude mice (2 × 10^6^ cells per mouse, *n* = 5 for each group). **h** The volumes of tumors were measured at 1–4 weeks after hypodermic injection and the growth rates of xenograft tumors upon shUBAC2 or shNC treatment were analyzed. **i** The weights of xenograft tumors were measured and analyzed after one month of hypodermic injection. **j** Ki67 staining were performed to show the proliferation index in xenograft tumors. The images were analyzed by calculating the integrated optical density per stained area (IOD/area). Data are presented as means ± SEM from three independent replicates or as means ± SEM of five mice from each group. **p < 0.01, ***p < 0.001 (Student’s *t* test).

In order to explore the function of UBAC2 in BC cells, we selected EJ and UMUC3 cells with the highest expression of UBAC2 for the further experiments. Meanwhile, four shRNAs targeting the coding region of UBAC2 (shUBAC2) were designed and stably transfected into EJ and UMUC3 cells with puromycin antibiotic selection. The efficiency of UBAC2 knockdown was detected by qRT-PCR and western blotting (Fig. 2a, b). We finally selected shUBAC2-1 and shUBAC2-3 for further studies because of their obvious knockdown effects. The cell cycle assay suggested that knockdown of UBAC2 resulted in cell cycle arrest at G0/G1 phase in EJ and UMUC3 cells (Fig. 2c). Besides, we found that the growth rate of both EJ and UMUC3 cells stably transfected with shUBAC2 decelerated significantly through CCK8 assay (Fig. 2d). Consistently, plate clone formation assay indicated that knockdown of UBAC2 remarkably suppressed the growth capability of EJ and UMUC3 cells (Fig. 2e). Nevertheless, the results from transwell migration assay and apoptosis assay showed that knockdown of UBAC2 did not affect the migration and apoptosis of EJ or UMUC3 cells (Fig. S1a, b).

To further explore the biological function of UBAC2 in vivo, nude mice were injected with EJ cells stably transfected with shUBAC2 or control plasmids. The results indicated that the growth rate and the weight of xenograft tumors derived from shUBAC2 stable transfectants decreased significantly compared with control group (Fig. 2f–i). Moreover, knockdown of UBAC2 significantly reduced the expression level of the proliferation marker Ki-67 (Fig. 2j). Collectively, these findings demonstrated that UBAC2 played an oncogenic role through promoting proliferation and cell cycle progression in BC cells.

### Knockdown of UBAC2 inhibits BC cells proliferation through increasing the expression of p27

**Fig. 3:**
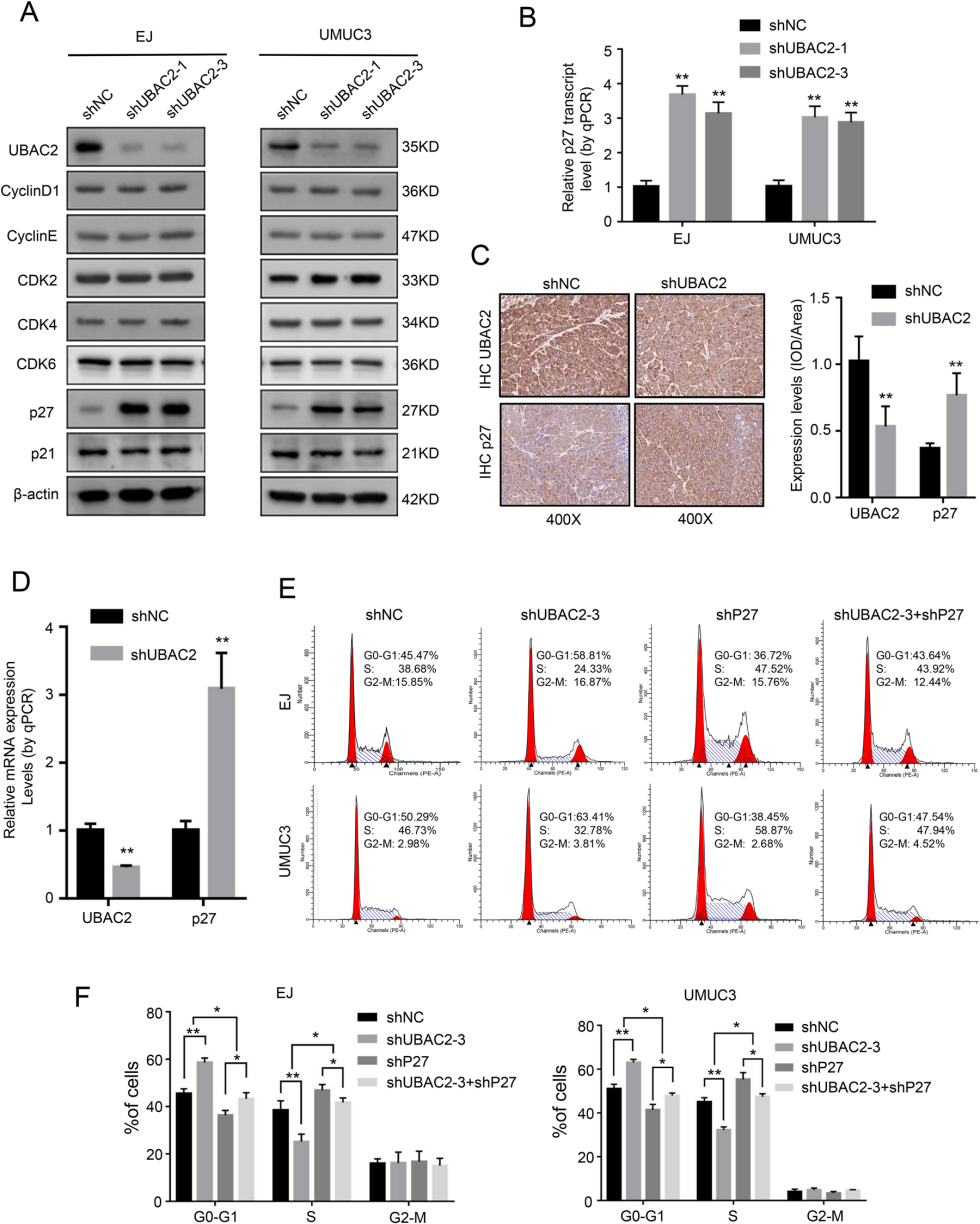
Knockdown of UBAC2 increases the expression of p27. **a** The expression levels of cell cycle related proteins (cyclin D1, cyclin E, CDK2, CDK4, CDK6, p27, and p21) were analyzed by western blotting after transfection with shUBAC2 or shNC in EJ and UMUC3 cell lines. β-actin was used as loading control. **b** The results from qRT-PCR showed that knockdown of UBAC2 significantly promoted the mRNA expression of p27 in EJ and UMUC3 cell lines. β-actin was used as loading control. **c** The protein expression level of UBAC2 and p27 in xenograft tumors was detected by immunohistochemistry (IHC) staining and analyzed by calculating the integrated optical density per stained area (IOD/area). **d** The mRNA expression level of UBAC2 and p27 in xenograft tumors from nude mice was determined by qRT-PCR. **e**, **f** Cell cycle distributions in shNC group, shUBAC2-3 group, shP27 group and shUBAC2-3 + shP27 group were presented by flow cytometry. Data are presented as means ± SEM from three independent replicates or as means ± SEM of five mice from each group. *p < 0.05, **p < 0.01, ***p < 0.001 (Student’s *t* test).

To determine the molecular mechanism of UBAC2 that promoted BC cells proliferation, we detected the potential interactions between UBAC2 and a series of key proteins related to cell cycle by western blotting assay. The key proteins included cyclin D1, cyclin E, CDK2, CDK4, CDK6, p27, and p21, and the results showed that p27 was the only upregulated protein in shUBAC2 stable transfectants (Fig. 3a). Meanwhile, knockdown of UBAC2 also induced the upregulation of p27 mRNA expression (Fig. 3b). Consistently, the efficiency of UBAC2 knockdown was also obvious in vivo, and we found that both protein and mRNA expression levels of p27 in xenograft tumors from shUBAC2 group were significantly higher than that from control group (Fig. 3c, d).

We further examined whether p27 was involved in shUBAC2-induced cell cycle arrest. The shRNAs targeting p27 (shP27) were obtained from our previous study^32^ and shP27-2 was finally selected for further studies owing to its better knockdown effect (Fig. S2a, b). We found that cell cycle progression was promoted by knockdown of p27 in BC cells (Fig. 3e, f). Furthermore, shUBAC2-induced cell cycle arrest was partially reversed by knockdown of p27 (Fig. 3e, f). Based on these results, we confirmed that knockdown of UBAC2 could suppress cell proliferation via increasing the expression of p27.

### UBAC2 can directly bind to circular RNA BCRC-3

**Fig. 4:**
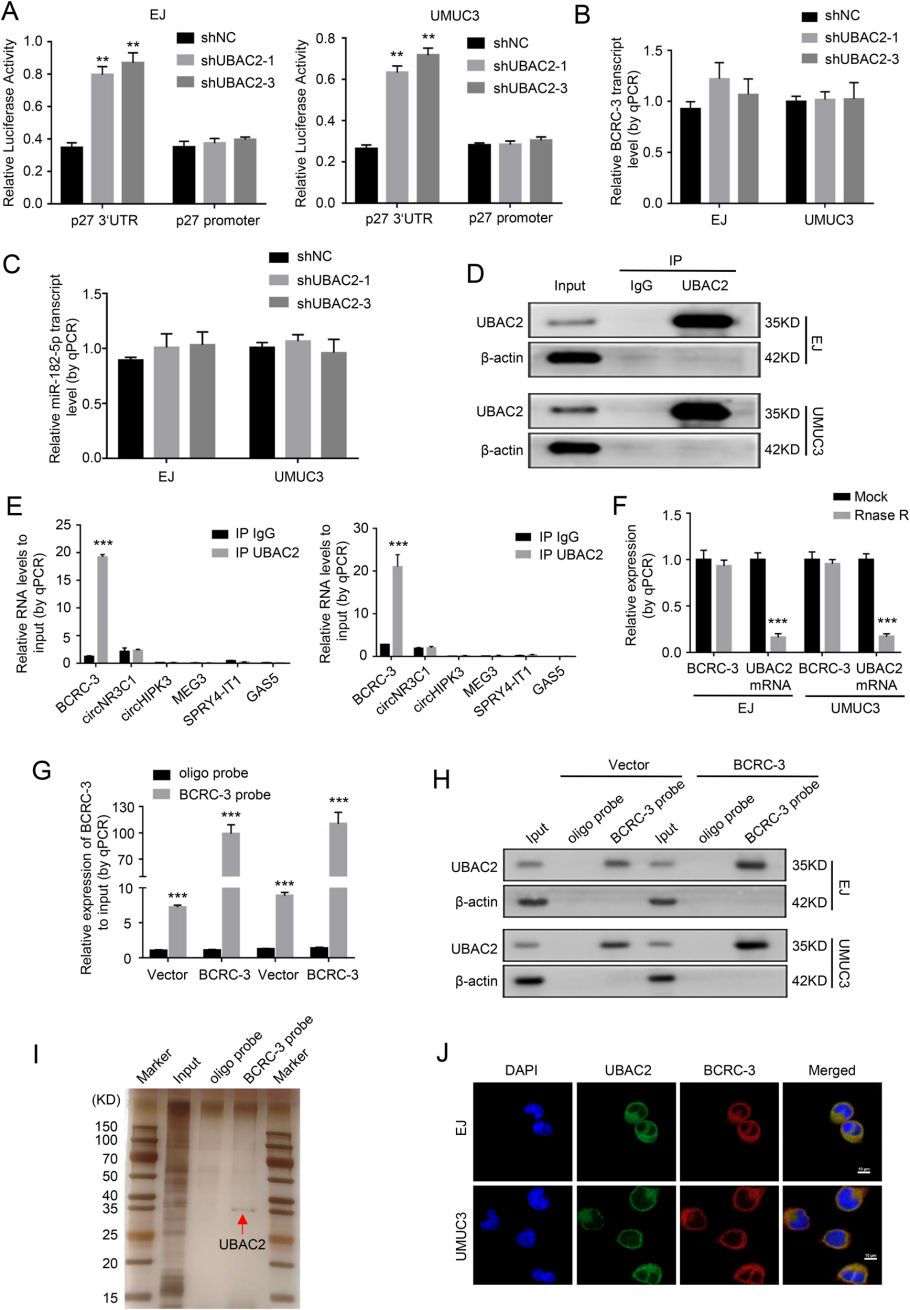
UBAC2 directly binds to BCRC-3. **a** The luciferase activity of p27 3’UTR and promoter after transfection with shUBAC2 or shNC in EJ and UMUC3 cell lines. **b**, **c** The results from qRT-PCR assay showed the expression levels of BCRC3 (**b**) and miR-182-5p (**c**) after transfection with shUBAC2 or shNC in EJ and UMUC3 cells. β-actin or U6 was used as loading control. **d** The efficiency of RNA immunoprecipitation (RIP) was detected by western blotting. **e** The expression levels of six noncoding RNAs in EJ and UMUC3 lysates were analyzed by qRT-PCR after UBAC2 immunoprecipitation assay. β-actin was used as negative control. Relative levels of six noncoding RNAs were normalized to input. **f** qRT-PCR analysis of the expression of BCRC-3 after RNase R treatment in lysates from UBAC2 immunoprecipitation assay. The expression of UBAC2 mRNA in input was used as positive control. **g** The efficiency of biotin-BCRC-3 pull-down assay was verified by qRT-PCR assay. β-actin was used as negative control. Relative level of BCRC-3 was normalized to input. **h** Western blotting assay analyzed the expression of UBAC2 in the EJ and UMUC3 lysates after biotin-BCRC-3 pull-down assay. **i** Silver staining was performed after SDS-polyacrylamide gel electrophoresis with the EJ lysate from pull down assay. **j** IF and FISH detection showed that UBAC2 and BCRC-3 were co-localized in the cytoplasm of BC cells. Nuclei was stained blue with DAPI. UBAC2 protein was stained green. BCRC-3 was stained red with cy3 (scale bar, 10 μm). Data are presented as means ± SEM from three independent replicate experiments. **p < 0.01, ***p < 0.001. (Student’s *t* test).

In order to explore how UBAC2 affected the expression of p27, the p27 3′-UTR and promoter luciferase reporter assays were performed. The results showed that knockdown of UBAC2 significantly amplified the activity of p27 3′-UTR in EJ and UMUC3 cells, while the activity of p27 promoter was not affected (Fig. 4a). Similarly, our previous studies demonstrated that BCRC-3 could promote the expression of p27 through binding with miR-182-5p and preventing it from blocking p27 3′UTR activity^32,37^. We next investigated whether UBAC2 affected the expression of BCRC-3 or miR-182-5p and then affected the expression of p27. Nevertheless, the results from qRT-PCR suggested that the expression levels of BCRC-3 or miR-182-5p were not significantly affected by knockdown of UBAC2 in EJ and UMUC3 cells (Fig. 4b, c). Meanwhile, we analyzed the expression of BCRC-3 and miRNA-182-5p in xenograft tumors by qRT-PCR and found the same results (Fig. S3a, b).

Then, we performed RIP to identify whether UBAC2 could directly bind to BCRC-3. The RIP efficiency was detected by western blotting (Fig. 4d) and the results from qRT-PCR showed that BCRC-3 was the only one that antiUBAC2 antibody abundantly immuno-precipitated in the six noncoding RNAs we have studied before, including BCRC-3, circNR3C1, circHIPK3, MEG3, SPRY4-IT1, and GAS5 (Fig. 4e). Meanwhile, RNase R was used to pretreat lysates from UBAC2 immunoprecipitation assay and the results from qRT-PCR showed that the BCRC-3 pulled down was circular (Fig. 4f). To further confirm the direct binding of UBAC2 and BCRC-3, we performed RNA pull-down assay and the pull-down efficiency was shown in Fig. 4g. The results from western blotting and silver staining suggested that UBAC2 was considerably pulled down by the BCRC-3 probe (Fig. 4h, i). Consistently, the merged images derived from IF and FISH detection demonstrated that UBAC2 and BCRC-3 were co-localized in the cytoplasm of BC cells, and the same results were found in EJ cells stably transfected with shUBAC2 (Fig. 4j and Fig. S4a, b). The above results indicated that UBAC2 could directly bind to BCRC-3 in BC cells.

### Knockdown of UBAC2 increases the expression of p27 through affecting the interaction of BCRC-3 with miR-182-5p in BC cells

**Fig. 5:**
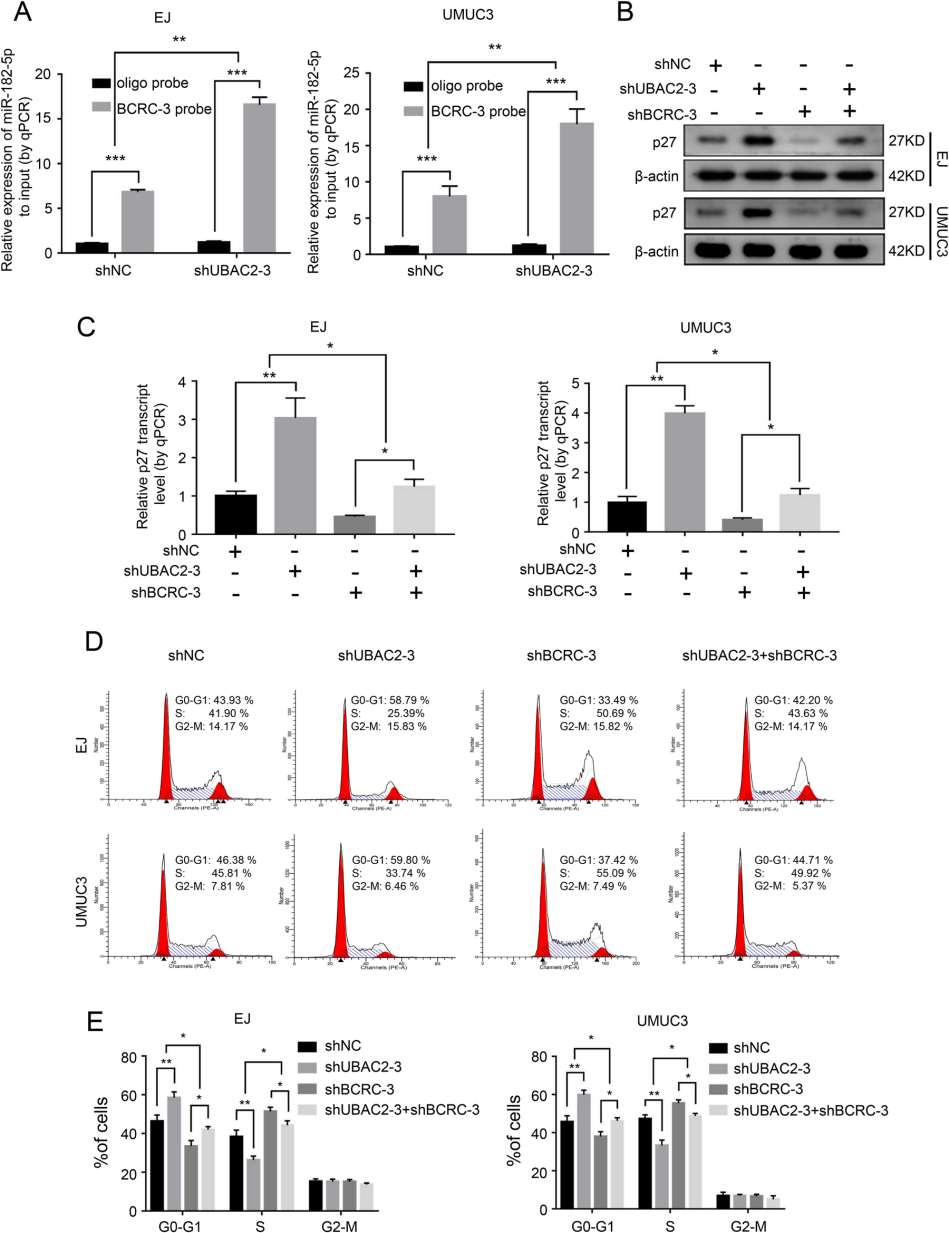
Knockdown of UBAC2 affects the interaction of BCRC-3 with miR-182-5p in BC cells. **a** The expression level of miR-182-5p was determined by qRT-PCR in EJ and UMUC3 transfected with shUBAC2-3 or shNC lysates after biotin-BCRC-3 pull-down assay. U6 was used as negative control. Relative level of miR182-5p was normalized to input. **b**, **c** The protein (**b**) and mRNA (**c**) expression levels of p27 in the EJ and UMUC3 after co-transfection with shUBAC2-3 and shBCRC3. β-actin was used as loading control. **d**, **e** Distribution of cell population in G1, S, and G2 phase in shNC group, shUBAC2-3 group, shBCRC3 group and shUBAC2-3 + shBCRC3 group. Data are presented as means ± SEM from three independent replicate experiments. *p < 0.05, **p < 0.01, ***p < 0.001 (Student’s *t* test).

**Fig. 6:**
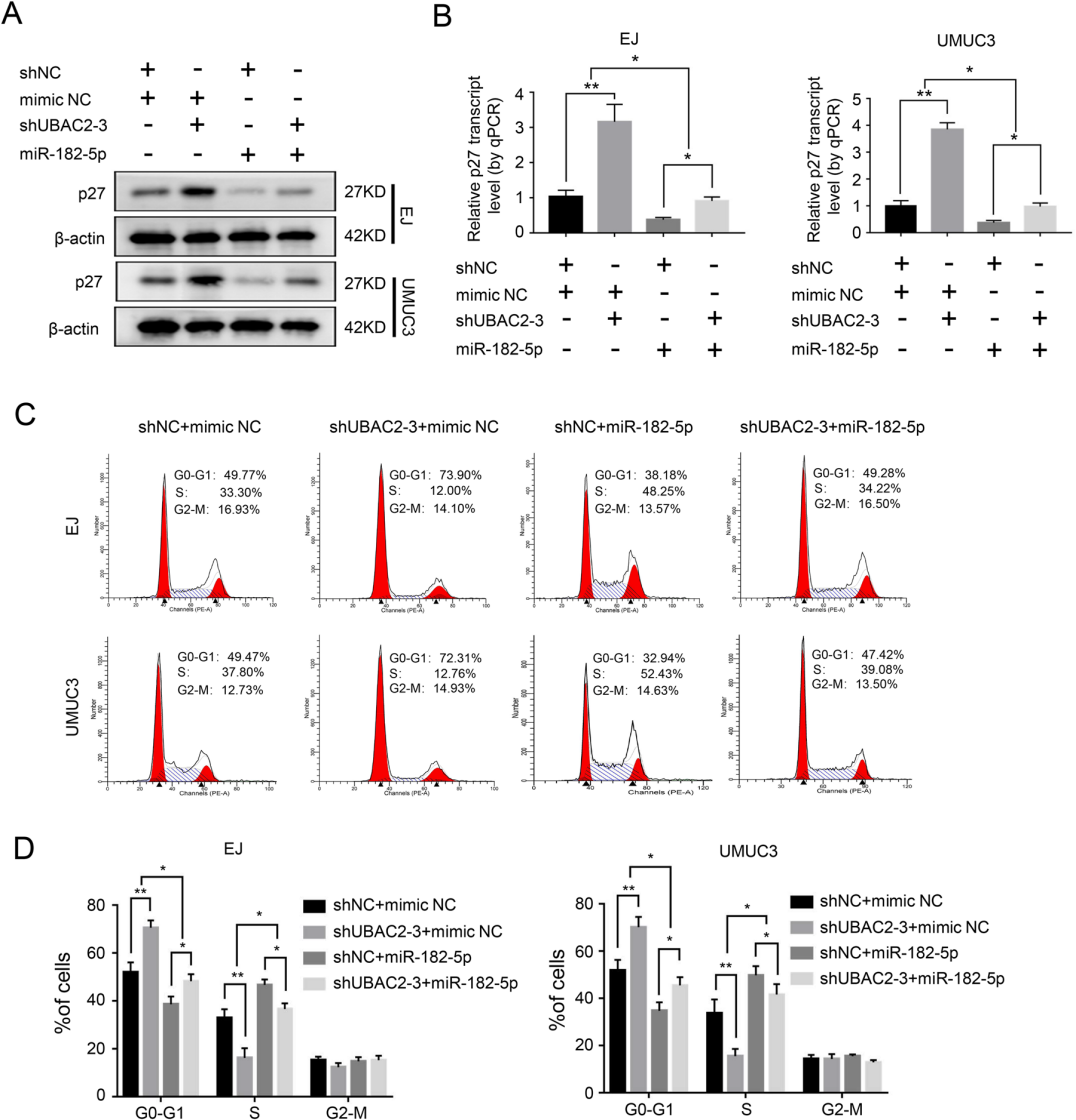
Overexpression of miR-182-5p partly reversed the effects of shUBAC2. **a**, **b** Western blotting (**a**) and qRT-PCR (**b**) analysis of the expression levels of p27 in the EJ and UMUC3 after co-transfection with shUBAC2-3 and miR-182-5p mimics. β-actin was used as loading control. **c**, **d** Cell cycle distributions in shNC + mimic NC group, shUBAC2-3 + mimic NC group, shNC + miR-182-5p group and shUBAC2-3 + miR-182-5p group were presented by flow cytometry. Data are presented as means ± SEM from three independent replicate experiments. *p < 0.05, **p < 0.01, ***p < 0.001 (Student’s *t* test).

**Fig. 7:**
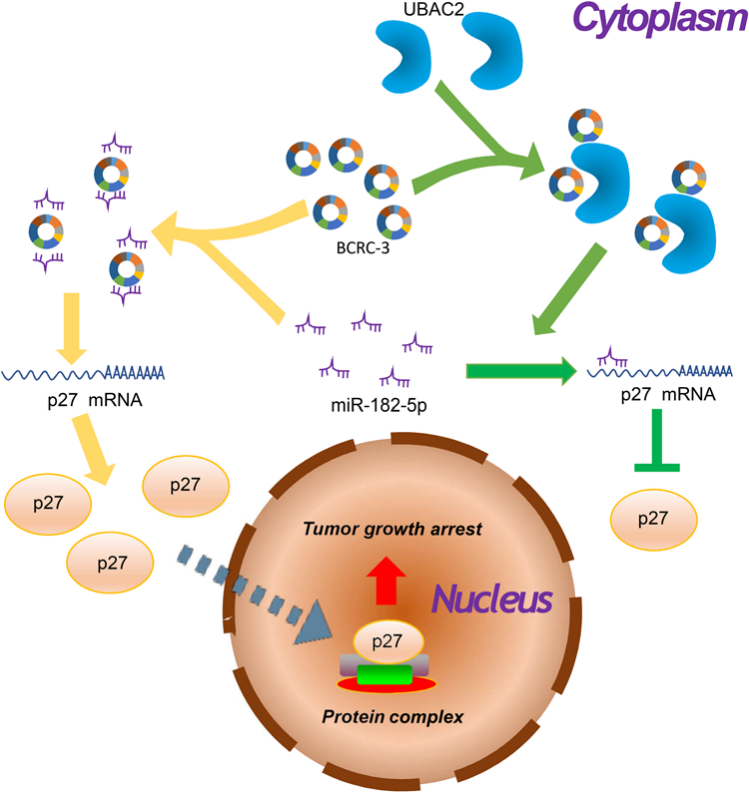
Graphical abstract. Graphical diagram of UBAC2 promoting bladder cancer proliferation through BCRC-3/miRNA-182-5p/p27 axis.

We further investigated whether the combination of UBAC2 and BCRC-3 affected the interaction of BCRC-3 with miR-182-5p by using pull-down assay. The results showed that the BCRC-3 probe pulled down significantly more miR-182-5p upon knockdown of UBAC2 in EJ and UMUC3 cells (Fig. 5a). Importantly, the increased effects of shUBAC2 on both the protein and mRNA expression levels of p27 were partly reversed upon knockdown of BCRC-3 in EJ and UMUC3 cells (Fig. 5b, c). The efficiency of BCRC-3 knockdown was shown in Fig. S2c, and we ultimately selected shBCRC-3-3 for further studies because of its better knockdown effect. Consistently, shUBAC2-induced cell cycle arrest was partly reversed upon knockdown of BCRC-3 (Fig. 5d, e). Moreover, ectopic expression of miR-182-5p partly reversed the increased expression of p27 and cell-cycle arrest induced by UBAC2 inhibition (Fig. 6a–d). Taken together, these results demonstrated that UBAC2 could regulate the expression of p27 through affecting the interaction of BCRC-3 with miR-182-5p in BC cells (Fig. 7).

## Discussion

The biological roles of RNA-protein interactions are pervasive^38^. Especially the effects of RNA binding proteins (RBPs) on RNAs, are key aspects of many cellular processes that go beyond the already established steps of the mRNA production and usage as information carriers, including splicing, polyadenylation, transport, stability and translation^38–41^. RBPs have been studied for decades, yet our understanding of RBPs is mainly limited to proteins with known or predicted RNA-binding domains (RBDs), which are considered to be the major proteins interacting with RNA^42^. Recently, 555 proteins constituting the mouse embryonic stem cells mRNA interactome were defined^42^. Strikingly, 216 of the 555 mRNA interactome proteins (39%) do not carry any known RBDs in mouse embryonic stem cells^42^. Additionally, more than 300 novel RNA-binding proteins were also defined in HeLa^43^ and HEK293^44^ cells and a lot of these proteins do not contain canonical RBDs. Recent study has demonstrated that TRIM25, a member of the Tripartite Motif (TRIM) family of E3 ubiquitin ligases without canonical RNA-binding domain, can act as a novel RNA-binding protein to directly interact with mRNA^45^. Furthermore, a growing knowledge of RBPs targets is shifting the attention towards non-coding RNAs, from RNAs involved in the translation machinery and its regulation (rRNAs, tRNAs, small interfering RNAs and miRNAs) to lncRNAs and circRNAs^46,47^. For instance, the cell cycle proteins cyclin-dependent kinase 2 (CDK2) and cyclin-dependent kinase inhibitor 1 (p21) can bind to circ-Foxo3, resulting in the formation of a ternary complex^28^. It has been reported that the E3 ubiquitin ligase CUL4A without RBD can interact with lncRNA uc.134 to form a RNP complex^48^. UBAC2 is also an ubiquitination related protein without RBD, and we demonstrate that UBAC2 can directly bind to BCRC-3 in the present study. These interactions could be caused by the fact that the tertiary structures of circRNAs results in greater protein binding capacity than those of linear RNA sequences^46,47^. Taken together, our study discovers that ubiquitin-related protein can bind to circRNA for the first time, which provides further evidence for the interaction between proteins and non-coding RNAs.

Circular RNAs (circRNAs), attracting great attentions for their closed continuous loop structure and potential value in clinical work, are produced from precursor mRNA (pre-mRNA) back-splicing of thousands of genes in eukaryotes. RBPs have been reported to regulate the biogenesis of circRNAs through direct binding to circRNAs^18,35,49–51^. For instance, the splicing factor Muscleblind (Mbl) regulates circRNA production from its own pre-mRNA through specific and direct binding to conserved muscleblind binding sites of circRNA (circMbl)^50^. The RBP Quaking (QKI) can regulate the formation of circRNAs by binding to two flanking introns and bring the circularized exons closer together, resulting in upregulation of circRNAs production in human epithelial–mesenchymal transition (EMT)^35^. In addition, RBPs also regulate the degradation of circRNAs. For instance, GW182, a key component of P-body and RNAi machine, plays a crucial role in the process of circRNAs degradation through mediating the interactions between circRNAs and circRNA decay factors^52^. However, we found that the expression level of BCRC-3 was not affected by UBAC2 in this study. Subsequently, we explored whether the function of BCRC-3 was affected by UBAC2, and found that UBAC2 could affect the inhibition of BCRC-3 on the proliferation of BC cells. On the other hand, previous studies have reported that circRNAs can affect the functions of proteins via direct interaction with them^23–26^. It has been reported that UBAC2 can negatively regulate the canonical Wnt signaling pathway in the lymphocytes by promoting the ubiquitin-mediated degradation of CTNNB1 and Wnt receptors FZD6 and LRP6^53^, and restrict trafficking of UBXD8 from the endoplasmic reticulum to lipid droplets^54^. Whether the function of UBAC2 are affected by BCRC-3, is deserved our further investigation. On the other hand, the regulatory mechanism of tumorigenesis and development is complex, and many regulatory pathways are intertwined^55^. One molecule may participate in multiple regulatory pathways^55^. In the future research, we will further explore ubiquitin related regulatory mechanism of UBAC2 in BC.

To date, numerous functions of circRNAs have been discovered, such as modulating parental gene expression^56,57^, regulating alternative splicing^50^, acting as miRNA sponges^20,58^, acting as protein sponges or protein scaffolds^26,59^, and so on. Among them, a well-defined function of circRNAs is binding with microRNAs (miRNAs) and sequestering them away from their target mRNAs as competitive^23^. In our previous study, we have identified that BCRC-3, circHIPK3, circNR3C1, and circ0001361 can act as “miRNA sponge” to regulate the progress of BC^32,36,60,61^. In these circRNAs, BCRC-3 inhibits BC cells proliferation through acting as “miRNA sponge” for miR-182-5p to promote the expression of p27^32^. In the present study, we found that p27 was also the effector molecule of UBAC2 in regulation BC cells proliferation. P27(Kip1) is a member of the KIP/CIP family of cyclin/cyclin-dependent kinase (CDK) inhibitors and regulates cell-cycle progression at the G1 to S phase transition. A lot of studies showed that the expression of p27 protein were reduced in many tumors, including colon cancer^62^, lung cancer^63^, prostate cancer^64^, BC^65^, breast cancer^66^, and some hematological malignancies^67^. In BC, induction of p27 is frequently found to be involved in inhibition of BC cells proliferation^68,69^ and decreased p27 expression is associated with poor overall and post-relapse survival^70^. The expression of p27 can be regulated by transcriptional and posttranscriptional mechanisms^37^. In this research, we demonstrated that UBAC2 participated in the post transcriptional regulation of p27 through affecting the interaction of BCRC-3 with miR-182-5p. Our findings further extend the upstream regulatory network of post transcriptional regulation of p27 in BC cells.

In summary, we demonstrate that both mRNA and protein levels of UBAC2 are upregulated in BC tissues and cell lines for the first time, and the expression of UBAC2 is significantly correlated with the overall survival of patients. Furthermore, we find that knockdown of UBAC2 can inhibit BC cells proliferation by increasing the expression of p27, and UBAC2 can directly bind with BCRC-3. Finally, our results show that UBAC2 can inhibit the expression of p27 through BCRC-3/miRNA-182-5p/p27 axis, thus promote the proliferation of BC cells. Our results not only explain the potential mechanisms related to UBAC2 in regulation BC cells proliferation, but they also provide a potential attractive target for diagnosis and therapy of BC.

## Supplementary information

Supplementary Information

Supplementary Fig 1

Supplementary Fig 2

Supplementary Fig 3

Supplementary Fig 4

Supplementary Table 1 and Table 2

Supplementary Table 3

Supplementary Table 4
