# Supplementary material for: UBAC2 promotes bladder cancer proliferation through BCRC-3/miRNA-182-5p/p27 axis
Source: Cell Death Dis. 2020 Sep 10;11(9):733. doi: 10.1038/s41419-020-02935-7 (PMC7484802; doi:10.1038/s41419-020-02935-7)
Supplement: Supplementary file 1 — Supplementary Information [file 41419_2020_2935_MOESM1_ESM.docx]

**Supplementary Information**

Summary: Supplementary Information contains four Supplementary figures and four Supplementary Tables. Supplementary figures are the supplementary information for results and discussion.

Supplementary Table 1 contains correlation between UBA2 expression and clinicopathological factors of 48 patients with BC from our hospital. Supplementary Table 2 contains correlation between UBA2 expression and clinicopathological factors of 407 patients with BC from TCGA database. Supplementary Table 3 contains all the primers, shRNAs target sequences and oligonucleotide probe sequences used in this manuscript. Supplementary Table 4 contains vector backbone of shRNAs used in this study. Supplementary Table 1 and 2 are provided as a file. Supplementary Table 3 and 4 are provided as independent files.

**Supplementary Figure Legends**

**Fig. S1. Knockdown of UBAC2 does not affect the migration** **and apoptosis of BC cells**

**a** The cell migration capability was evaluated by the transwell migration experiments in shUBAC2 cells and corresponding control cells. Data are presented as mean ± SEM from three independent replicates. P>0.05 compared with control cells (Student’s t test). **b** Cell apoptosis rates in shUBAC2 cells and corresponding control cells were analyzed by ﬂow cytometry. Data are presented as mean ± SEM from three independent replicates. P>0.05 compared with control cells (Student’s t test).

**Fig. S2.** **Knockdown efﬁciency of p27 and BCRC-3**

**a**, **b** The efﬁciency of p27 knockdown in EJ and UMUC3 cells was detected by qRT-PCR(**a**) or western blotting assay(**b**). β-actin was used as loading control. **c** The efﬁciency of BCRC-3 knockdown was obtained from qRT-PCR. β-actin was used as loading control. Data are presented as mean ± SEM from three independent replicates. **P<0.01 compared with control cells (Student’s t test).

**Fig. S3.** **The** **expression of BCRC-3 and miRNA-182-5p in xenograft tumors.**

**a**, **b** The mRNA expression levels of BCRC-3 and miR-182-5p in xenograft tumors from nude mice were determined by qRT-PCR. β-actin or U6 was used as loading control. Data are presented as means ± SEM of 5 mice from each group. P>0.05 compared with control group (Student’s t test).

**Fig. S4.** **The** **results of IF and FISH**

**a** IF and FISH assay were performed to showed the location of UBAC2 and BCRC-3 in EJ cells stably transfected with shUBAC2. Nuclei was stained blue with DAPI. UBAC2 protein was stained green. BCRC-3 was stained red with cy3(scale bar, 10 μm). **b** IF and FISH assay were performed by IgG and oligo probe. Nuclei was stained blue with DAPI. IgG protein was stained green. Oligo probe was stained red with cy3 (scale bar, 10 μm).
