# Supplementary figures and images for: UBAC2 promotes bladder cancer proliferation through BCRC-3/miRNA-182-5p/p27 axis
Source: Cell Death Dis. 2020 Sep 10;11(9):733. doi: 10.1038/s41419-020-02935-7 (PMC7484802; doi:10.1038/s41419-020-02935-7)

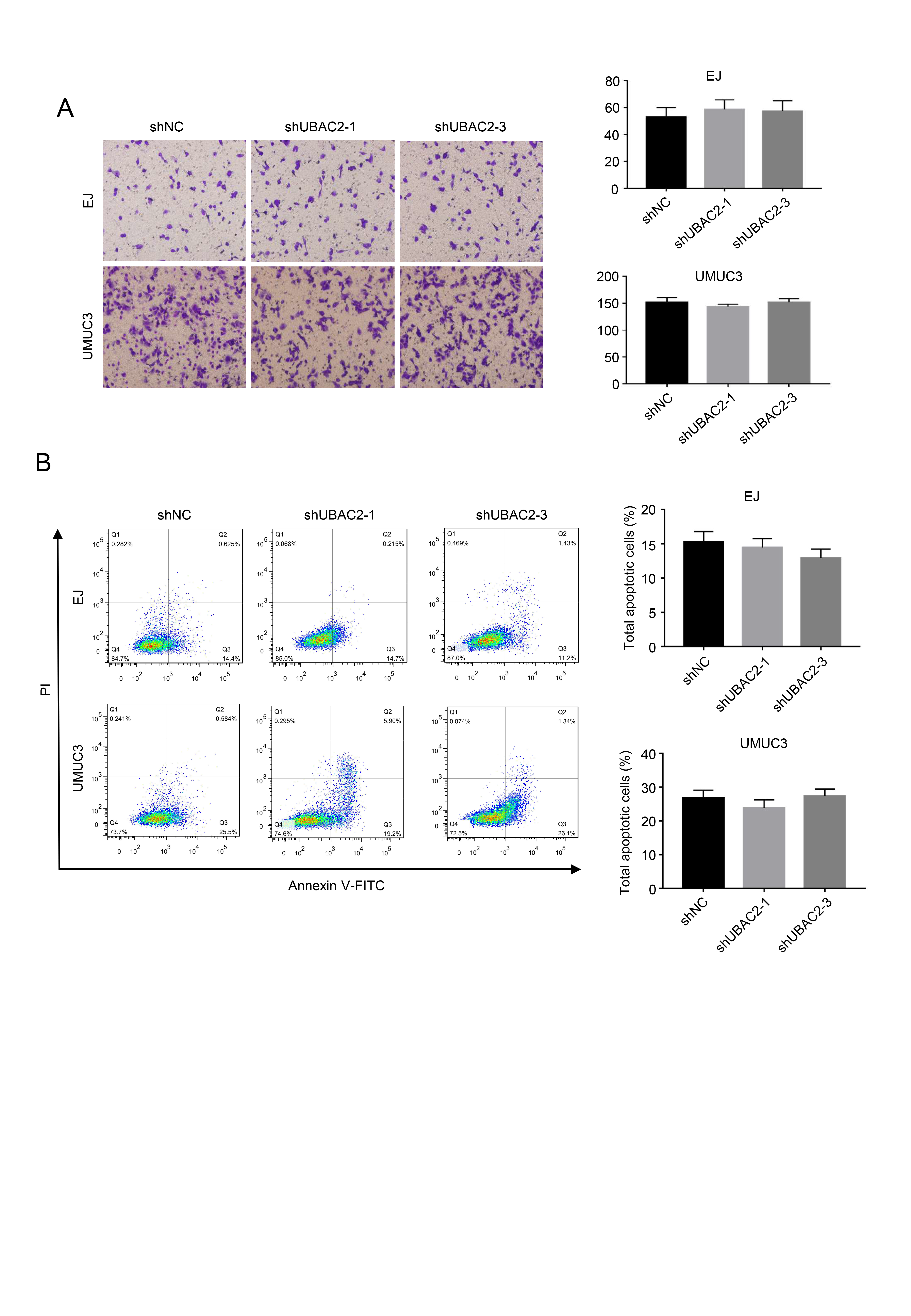

Supplement: Supplementary file 2 — Supplementary Fig 1 [file 41419_2020_2935_MOESM2_ESM.tif]

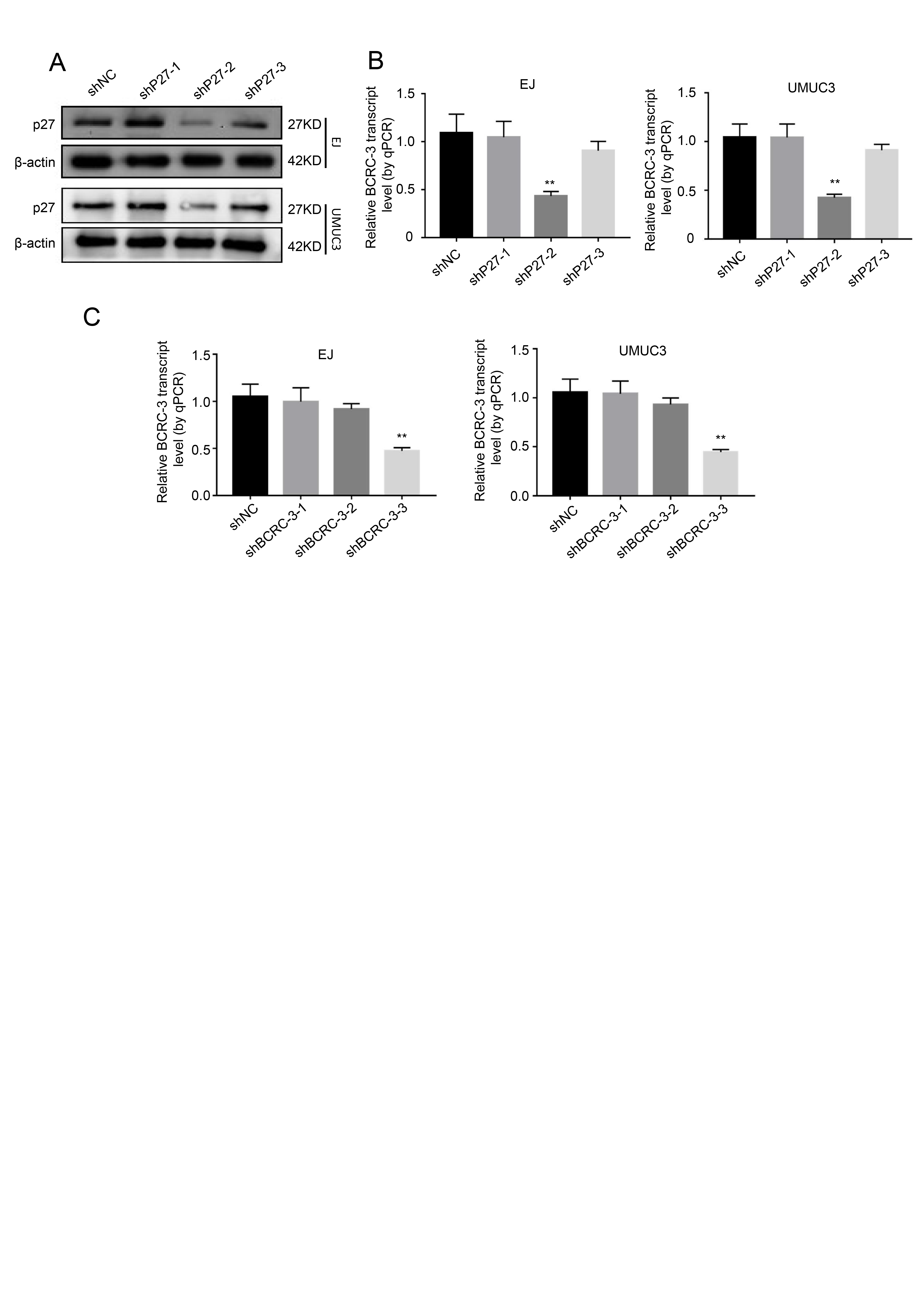

Supplement: Supplementary file 3 — Supplementary Fig 2 [file 41419_2020_2935_MOESM3_ESM.tif]

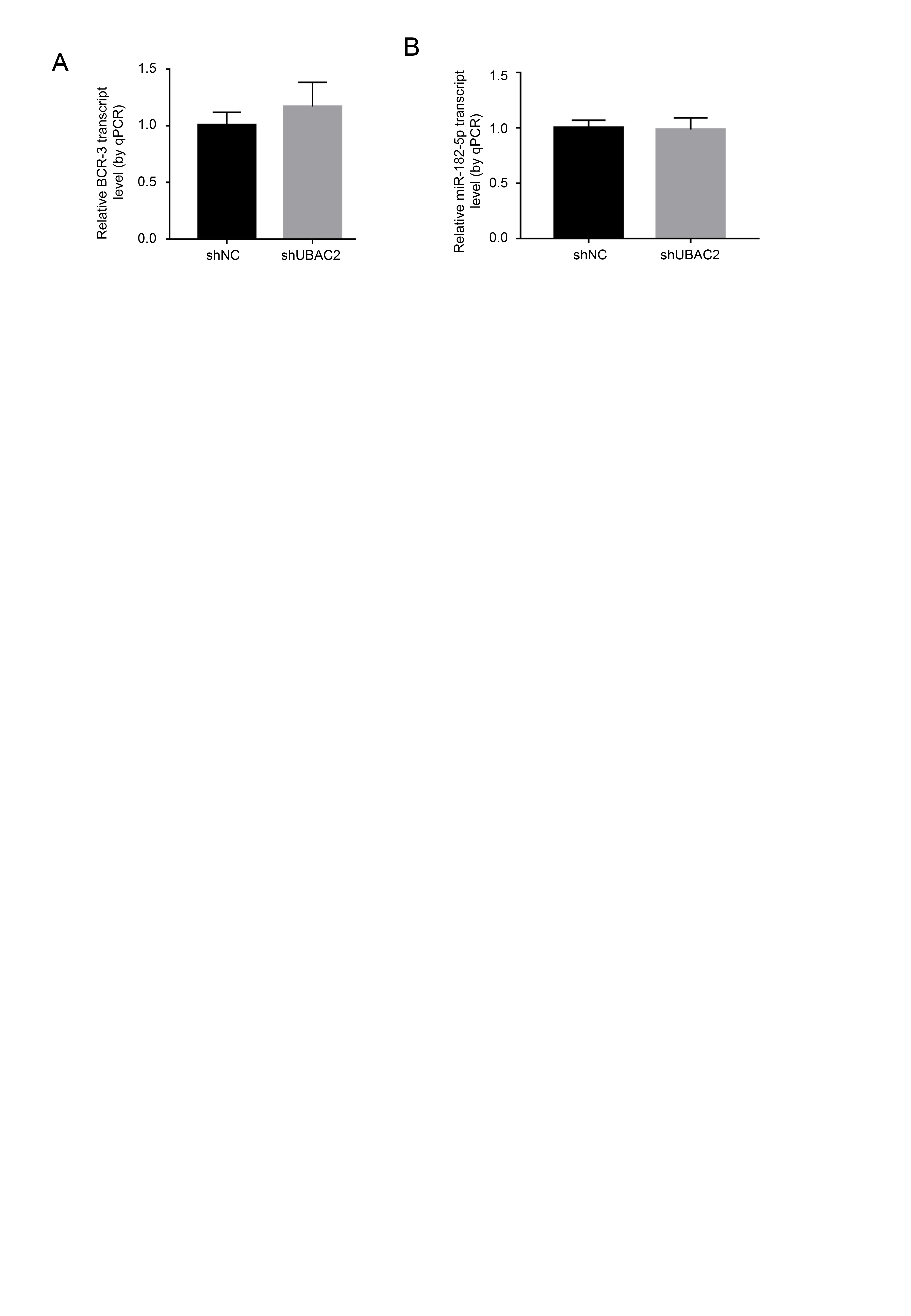

Supplement: Supplementary file 4 — Supplementary Fig 3 [file 41419_2020_2935_MOESM4_ESM.tif]

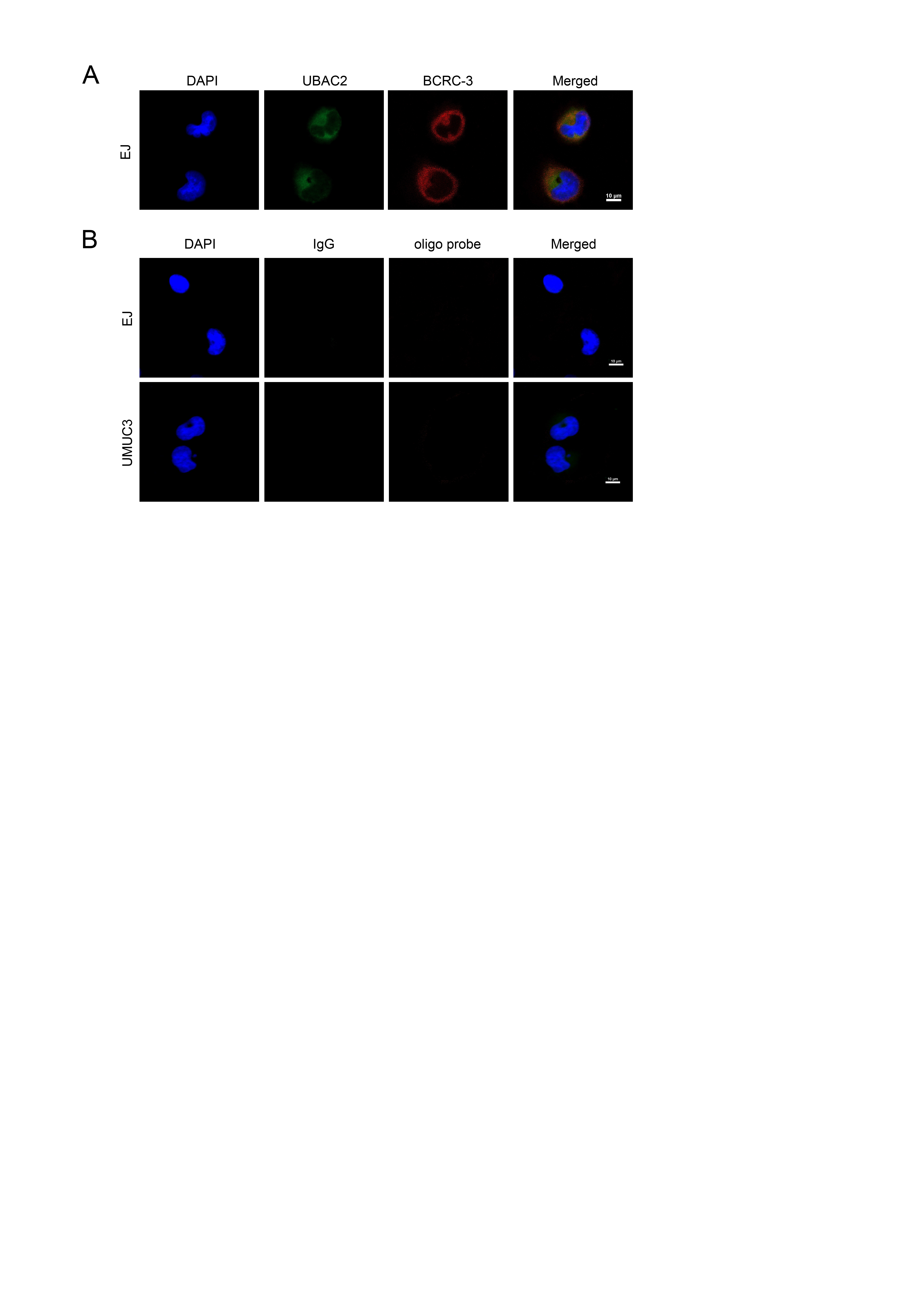

Supplement: Supplementary file 5 — Supplementary Fig 4 [file 41419_2020_2935_MOESM5_ESM.tif]
