# Supplementary material for: UBAC2 promotes bladder cancer proliferation through BCRC-3/miRNA-182-5p/p27 axis
Source: Cell Death Dis. 2020 Sep 10;11(9):733. doi: 10.1038/s41419-020-02935-7 (PMC7484802; doi:10.1038/s41419-020-02935-7)
Supplement: Supplementary file 6 — Supplementary Table 1 and Table 2 [file 41419_2020_2935_MOESM6_ESM.docx]

**Table 1 Correlation between UBAC2 expression and clinicopathological factors in bladder cancer (Data from our hospital, n=48).**

| Parameters | Group | Cases | UBAC2 expression | | | | | P  Value |
| --- | --- | --- | --- | --- | --- | --- | --- | --- |
|  |  |  | Low | % | | High | % |  |
| Gender | Male | 40 | 11 | 27 | 29 | | 73 | 0.8871 |
|  | Female | 8 | 3 | 37 | 5 | | 63 |  |
| Age(years) | <55 | 9 | 3 | 33 | 6 | | 67 | 0.9190 |
|  | ≥55 | 39 | 11 | 28 | 28 | | 72 |  |
| Tumor stage | pTa-T1 | 25 | 7 | 28 | 18 | | 72 | 0.8529 |
|  | pT2-T4 | 23 | 7 | 30 | 16 | | 70 |  |
| Tumor size | <3.0 cm | 21 | 7 | 33 | 14 | | 67 | 0.5754 |
|  | ≥3.0cm | 27 | 7 | 26 | 20 | | 74 |  |
| Grade | Low | 8 | 2 | 25 | 6 | | 75 | 0.8871 |
|  | High | 40 | 12 | 30 | 28 | | 70 |  |
| Lymph node  metastasis | Absent | 42 | 11 | 26 | 31 | | 74 | 0.4714 |
|  | Present | 6 | 3 | 50 | 3 | | 50 |  |

P < 0.05 represents statistical significance (Chi-square test).

**Table 2 Correlation between UBAC2 expression and clinicopathological factors in bladder cancer（Data from TCGA, n=407）**

| Parameters | Group | Cases | UBAC2 expression | | | | P  Value |
| --- | --- | --- | --- | --- | --- | --- | --- |
|  |  |  | Low | % | High | % |  |
| Gender | Male | 301 | 149 | 49.50 | 152 | 50.50 | 0.7985 |
|  | Female | 106 | 54 | 50.94 | 52 | 49.06 |  |
| Age(years) | <55 | 43 | 22 | 51.16 | 21 | 48.84 | 0.8585 |
|  | ≥55 | 364 | 181 | 49.73 | 183 | 50.27 |  |
| Tumor stage | T1-T2 | 134 | 68 | 50.75 | 66 | 49.25 | 0.8059 |
|  | T3-T4 | 273 | 135 | 49.45 | 138 | 50.55 |  |
| Grade | Low | 24 | 12 | 50.00 | 12 | 50.00 | 0.9901 |
|  | High | 383 | 191 | 49.87 | 192 | 50.13 |  |
| Lymph node  metastasis | Absent | 187 | 99 | 52.94 | 88 | 47.06 | 0.2544 |
|  | Present | 220 | 104 | 47.27 | 116 | 52.73 |  |

P < 0.05 represents statistical significance (Chi-square test).
