# Supplementary material for: UBAC2 promotes bladder cancer proliferation through BCRC-3/miRNA-182-5p/p27 axis
Source: Cell Death Dis. 2020 Sep 10;11(9):733. doi: 10.1038/s41419-020-02935-7 (PMC7484802; doi:10.1038/s41419-020-02935-7)
Supplement: Supplementary file 8 — Supplementary Table 4 [file 41419_2020_2935_MOESM8_ESM.docx]

**
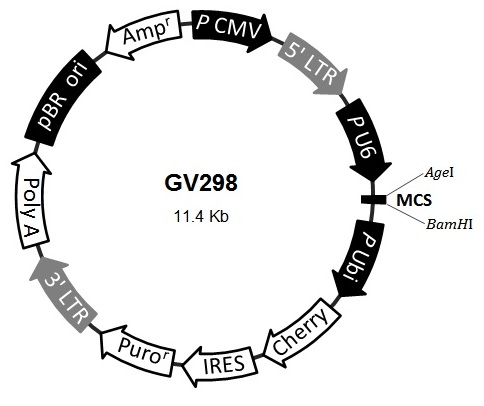
**

**Vector backbone of UBAC2 and BCRC-3 shRNAs**

The shRNAs targeting UBAC2 and BCRC-3 were synthesized and digested with restriction enzymes *Age* I and

*BamH* I, and then ligated to the restriction sites of GV298 vector.
